# The psychosocial needs of patients who have chronic kidney disease without kidney replacement therapy: a thematic synthesis of seven qualitative studies

**DOI:** 10.1007/s40620-022-01437-3

**Published:** 2022-09-01

**Authors:** Christina Seery, Sarah Buchanan

**Affiliations:** 1 Department of Psychology, Mater Misericordiae University Hospital, Dublin, Ireland; 2 UCD Department of Psychology, University College Dublin, Dublin, Ireland

**Keywords:** Psychosocial needs, Chronic kidney disease, Chronic kidney disease without kidney replacement therapy, Psychosocial support

## Abstract

**Background:**

Limited quantitative data suggests that patients who have chronic kidney disease without kidney replacement therapy (CKD without KRT) may present with psychosocial needs just as patients who have acute kidney injury and are treated by dialysis (AKI stage 3D) do. This systematic review aims to synthesise qualitative research on patients’ experiences of CKD without KRT to provide further insight into patients’ experience of the healthcare they receive and simultaneously, their psychosocial needs, to inform the development of appropriate psychological interventions.

**Methods:**

The review followed ENTREQ guidelines. PubMed/MEDLINE, PsycINFO, EMBASE and CINAHL were searched in July and August 2021. Qualitative studies in English on the experiences of CKD without KRT care were included in the review. Thematic synthesis was conducted on the findings of the included studies.

**Results:**

The search identified 231 articles for screening. Eight studies met the inclusion criteria, and one was excluded at the quality assessment stage. The final seven articles [*n* = 130 patients] were analysed. Five themes on psychosocial needs were developed: addressing patients’ CKD-related educational needs, supporting the patient’s relationships, honouring the patient’s need for control, adjusting to change, and recognising fear of disease and treatment.

**Discussion:**

This review highlights the range of psychosocial needs of patients who have CKD without KRT. There are numerous intervention options that clinicians may develop that could benefit patients and address multiple needs, such as group educational programmes.

**Graphical abstract:**

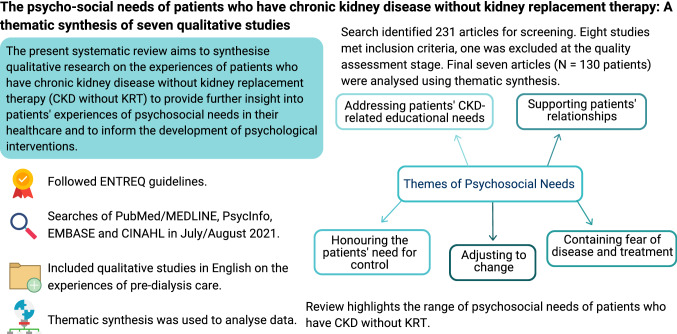

**Supplementary Information:**

The online version contains supplementary material available at 10.1007/s40620-022-01437-3.

## Background

The UK’s National Institute for Health and Care Excellence (NICE) guidelines on chronic kidney disease (CKD) recommend considering the psychological aspects of coping with CKD and offering access to support [1]. These recommendations echo practitioners’ and patients’ views on kidney failure [2, 3]. A systematic review recently identified the psychosocial needs of patients who have kidney failure, the majority of whom were dialysed or had received a transplant, highlighting a range of areas patients may need support with [4]. Qualitative findings indicate that patients experience substantial emotional burden with their illness, a complicated relationship with their treatment, fear of discussing their emotional difficulties with staff [5] and struggling to adapt to the invisibility and intangibility of kidney disease [6]. Given the high need for psychosocial support in patients who have kidney failure, there may be similar needs for patients who have CKD and are not receiving kidney replacement therapy (CKD without KRT), which may reduce psychological risk, and subsequent mortality [7] after their illness has progressed.

A range of psychosocial factors, such as anxiety, depression, psychological distress, and sleep disturbances, can have a bi-directional influence on chronic health conditions. It is vital to consider the patient holistically by addressing medical and psychological needs, to improve quality of life and cope with illness symptoms [7]. Quantitative findings have highlighted several potential targets in psychosocial interventions for patients who have CKD without KRT that may significantly affect patient outcomes. Many patients who have CKD without KRT have reported experiencing symptom burden and an impact on their health-related quality of life [9] and levels of elevated anxiety symptoms, comparable with patients who have acute kidney injury, stage 3, and are treated by dialysis (AKI stage 3D) [10]. Patients at a pre-dialysis stage also demonstrate only modest knowledge of CKD [11]. This is pertinent as multidisciplinary CKD without KRT education can reduce mortality and incidence of dialysis [12]. Therefore, addressing psychosocial needs within CKD without KRT care can mitigate later complications and improve the patient’s current quality of life.

The present review aims to synthesise qualitative studies on the experiences of patients who have CKD without KRT to identify their psychosocial needs. Qualitative research in nephrology can provide insights into patients’ experiences, values and priorities to inform practice and policies [13]. The experiences of patients, as described in qualitative studies, can be used to provide a deeper understanding of psychosocial needs at a CKD without KRT stage. By synthesising qualitative findings, this review can offer a patient-centred perspective to supplement quantitative data on the psychological well-being of patients who have CKD without KRT and inform the development of psychosocial supports.

## Methods

This systematic review was pre-registered on PROSPERO (CRD42021277376) and employed thematic synthesis, which aims to identify high-order themes [14] to develop descriptive or analytic themes to inform recommendations and guidelines [15], which enabled the identification of psychosocial needs. The reporting of this review followed the Enhancing Transparency in Reporting the Synthesis of Qualitative Research checklist [16], an alternative to the PRISMA guidelines, suitable for systematic reviews of qualitative research.

### Selection criteria and literature search

The search was pre-planned, and terms were selected with support from an evidence synthesis librarian and using the included databases’ thesauruses. Searches of PubMed/MEDLINE, PsycINFO, EMBASE and CINAHL were carried out in July and August 2021. Google Scholar was also reviewed for any articles that may have been missed in the official databases. Relevant references in systematic reviews that appeared in the search were included at the screening stage. The bibliographies for the final selection were reviewed for any additional studies. Qualitative studies in English that employed interviews, focus groups or observations about CKD without KRT were included. Participants must have been patients that had CKD without KRT or studies with patients who, at the time of the research, had AKI stage 3D reflecting on their care before kidney replacement therapy (KRT). Studies with non-patients, paediatric patients and mixed patient populations (unless they clearly identified data/themes about participants at a CKD without KRT stage) were excluded.

Following the database search, both authors screened the titles and abstracts of all articles before reviewing the full texts of articles against inclusion and exclusion criteria. Any discrepancies were discussed until consensus was achieved.

### Data extraction and quality assessment

The first stage of data extraction was to identify the contextual details of each study. All text labelled under “Results” was extracted. Extraction was conducted by CS and reviewed by SB. The Consolidated Criteria for Reporting Qualitative Studies (COREQ) [17] guided the quality assessment of included articles. Both reviewers separately evaluated the methods of each study using the COREQ before discussing their assessments to reach a consensus. The online supplementary material provides a summary of this assessment and reviewer conclusions for each study.

A measure called the Confidence in the Evidence from Reviews of Qualitative Research (CERQual) [18] was used to assess each review’s findings. The CERQual is used to determine confidence or if findings are a reasonable representation of the phenomenon of interest and a summary of qualitative findings. The full CERQual assessment is provided in the online supplementary material.

### Data synthesis

All extracted data were copied verbatim into Nvivo. Both authors independently coded each line of text before reviewing each study to check the consistency of interpretation and review codes where necessary. Codes were generated throughout the analysis of all studies. Text was analysed by assigning it a code or by creating a new code if none reflected its content. Descriptive themes were inductively developed collaboratively, and subsequently the analytical themes, in line with the research objectives [14].

## Results

### Results of the search

**Fig. 1:**
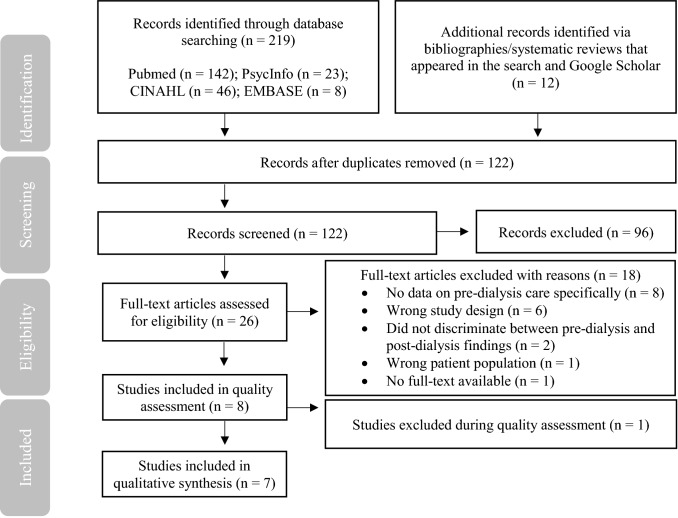
PRISMA diagram

The database search identified 231 studies, with eight studies meeting the inclusion criteria (see Fig. 1 for PRISMA diagram). One study [19] was excluded at the quality assessment stage due to significant methodological limitations and a lack of evidence to support stated themes. Seven studies were included in the final analysis.

Five studies used a sample of patients who had CKD without KRT (*n* = 62) [20–24], one study recruited patients who had AKI stage 3D (*n* = 47) [25], and one study gathered data from patients with differing stages of CKD [26]. The data of 21 patients that were not receiving KRT were included [26]. In total, the synthesis included 130 patients with CKD. A complete list of study characteristics can be found in Table 1.

**Table 1:** Characteristics of included studies

Study	Country	No. of participants	Age	CKD Information	Conceptual Methodological Framework	Qualitative Data Collection	Context	Analysis	Topic
Beanlands et al. [20]	Canada	11	19–39	CKD without KRT; category G1-3 glomerular-based CKD	Not reported	Focus groups	Focus groups with women ages 18–40, conducted in a conference room at the hospital where they received care	Content analysis	Explore issues young women are dealing with in relation to living with CKD and identify strategies they use to manage their illness
Iles-Smith [21]	United Kingdom	10	37–73	CKD without KRT Attending CKD without KRT clinic for 3 to 35 months	Not reported	Semi-structured interviews	Participants were intentionally interviewed before starting dialysis and prior to Tenckhoff catheter insertion or fistula formation	Thematic analysis	Perceptions, expectations and experiences of patients prior to starting dialysis therapy
Jennette et al. [25]	United States	47	21–80	Patients receiving peritoneal dialysis and haemodialysis Patients who have AKI stage 3D and time on dialysis ranged from less than 1 year to 9 years	Mixed methods, grounded theory	Focus groups	Recruited by nephrology social workers in dialysis clinics	Thematic analysis	Perspectives on methods for educating newly diagnosed patients about kidney replacement therapy; perceptions of their disease at onset and how these perceptions may influence treatment choices
Lissanu et al. [22]	United States	23	18–75	CKD without KRT	Not reported	Semi-structured interviews	Interviewers were also minorities; participants recruited from nephrology clinics or inpatient care	Not specified (identified themes and used frequency analysis)	Sought to understand African American patients’ knowledge of their CKD and ways they managed or prepared for illness progression
Tong et al. [26]	Australia	21 CKD without KRT, 63 in total	20–69	Mixed sample of patients with CKD without KRT, AKI stage 3D, CKD G1T–G5T	Not reported	Focus groups	Focus groups were conducted in nonclinical settings, in absence of physicians	Thematic analysis	CKD experiences and the impact of CKD treatment on lives of patients
Tweed and Ceaser [23]	United Kingdom	9	29–69	CKD without KRT Participants had been advised they were likely to require kidney replacement therapy within a time frame of 2–18 months	Phenomenology	Semi-structured interviews	Interviews were conducted in a convenient location for the participant, with both authors present to ask additional questions	Interpretative Phenomenological Analysis	Decision-making processes for kidney replacement therapy choices
Walker et al. [24]	United Kingdom	9	63–93	CKD without KRT (stage 4) Attending CKD without KRT clinic for 11–108 months	Not reported	Semi-structured interviews	Interviews were conducted in a kidney outpatient department	Thematic analysis	Older patients’ experiences of integrating lifestyle changes

### Synthesis

We identified five themes relating to psychosocial needs:

- Addressing the patient’s CKD-related educational needs
- Supporting the patient’s relationships
- Honouring the patient’s need for control
- Adjusting to change
- Recognising fear of disease and treatment

**Fig. 2:**
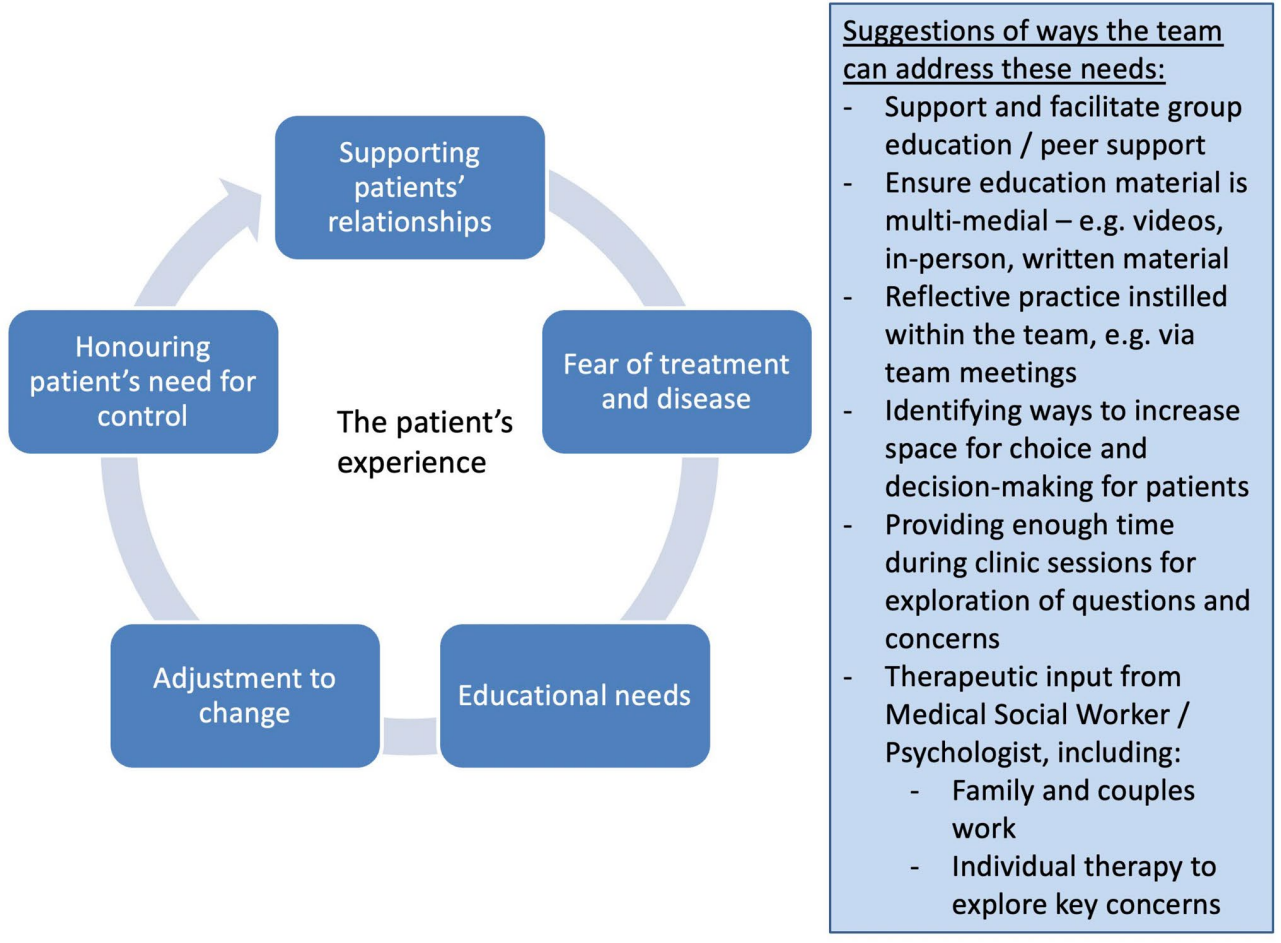
Suggestions of how the multidisciplinary team might support psychosocial needs in patients who have CKD without KRT

A summary of qualitative findings with illustrative quotes and a CERQual assessment is provided in Table 2. Figure 2 provides an overview of how the themes can be understood in terms of the multidisciplinary team.

**Table 2:** Summary of Qualitative Findings with CERQual Assessment

Theme	Summary of review finding	Recommendations for patient interventions	Studies contributing to the review finding	CERQual assessment of level of confidence in the evidence	Explanation of CERQual assessment	Illustrative Quotes
Addressing the patients’ CKD-related educational needs	Patients felt that they did not receive sufficient information about their healthcare, or that it was overly confusing. They had additional questions left unanswered and wanted additional time with their healthcare team to discuss their concerns	Communication training and group-based education could support educational needs	[20–26]	Moderate confidence	Minor concerns regarding methodological limitations, coherence and adequacy	“Having information would be helpful and useful… it empowers you… And also, like understanding it right away in part I think takes away part of the fear of what it is [20] “I didn’t know what any of it (dialysis) involved, … nobody told me,” [21] “I’d like somebody to have spent an hour or so, or whatever it takes and explain exactly everything about it … why it has to be that way” [21] “You know they got a certain number of people got to go on dialysis at the hospital. Why not take me into a conference room and sit down, show me some pictures, have someone come and explain what's going on?” [25] “When my doctor sent me down there, they just put me in a room, showed me a film about people on dialysis, even my doctor didn't sit down and talk to me and tell me what was going on.” [24] “They understood where I was coming from straight away wanting CAPD. They could see why I could chose it, that it did fit in with my life better and, if anything, I'd made up my mind anyway and after I was talking to them, it confirmed it.” [23]
Supporting the patients’ relationships	***Healthcare professionals.*** Patients valued attentive, non-judgemental medical care but sometimes found this was lacking, creating a sense of distrust and disrespect	Engaging patients in educational conversations about CKD and creating a space to listen and answer questions could improve their relationships with healthcare professionals	[20, 22–26]	Moderate confidence	Minor concerns regarding methodological limitations, coherence and relevance and moderate concerns regarding adequacy	“And everybody always- were just were really concerned and spent time with me and explained and whatever, so it was really a partnership in making sure that I stayed healthy... I liked how they coordinated and they’d be like, ‘ok, you’re going to have to see three doctors, but let’s just poke you once.’ So then I would bring the requisition to everybody and then they would fill out one form so that I’m [not] going getting blood work every, you know, day...” [20] “He should take a little more interest, rather than just fob me off and say, go see another doctor, because it may be related or not related to the kidney problem.” [26] “I think that one of the things we all have felt that I don't think anyone has said, is the problem of people meaning to tell us or give us a misconception of how you are going to feel.” [25]
	***Peers.*** Peers were an educational and supportive resource to patients	Group interventions or peer support could be a potential effective patient intervention				“When she rolled the sleeves up on her blouse [to show fistula] I won't say I couldn't believe what I saw, that's not true. It was almost like watching a horror film…” [23] [speaking to another participant] “You were the one talked me into going on PD. She said, ‘There's someone I want you to talk to.’ You happened to be in the centre one day when I was on the other side.” [25] “The problem is you think you’re on your own.” (Tong et al., 2009) “Somewhere people can go and discuss it when they’re not at end stage, when they’re just living and managing to keep them living” [26]
	***Family and friends.*** Support from family and friends was important to patients, influencing their illness-related decision making. Patients felt conscious of burdening their loved ones with their illness	Clinicians could consider offering family/systemic support to foster open communication and curiosity about the challenges the patient faces				“I use three different people as sounding boards. 'P', who's got multiple sclerosis, so get his point of view and 'E' whose son died of kidney failure and then 'S', who's perfectly fit.” [23] “As you can imagine that when my wife does cooking. She doesn’t use tomatoes at all…… and I feel guilty about that, I do tell them.” [24] “I take about at least ten a day. You know, ten in the morning and two in the evening. [Interviewer: Is it hard to keep straight what you’re taking?] Yeah. I have, you know, my wife makes it up. You know, I have the pill box.” [22]
Honouring the patients’ need for control	At times, patients felt like they lacked control of their illness and treatment, creating a feeling of helplessness	Feeling listened to, educated on their treatment and addressing symptoms using psychosocial support could foster self-efficacy	[20, 21, 23, 25, 26]	Moderate confidence	Minor concerns regarding methodological limitations, coherence, adequacy and relevance	“If I’m going to feel this bad for the rest of my life, do I just want to end it now?” [26] “Nothing’s going to make you better, make you happy, but you do have to go on.” [20] “I don’t know because Jesus knows.” [21] “I had very high expectations and I don’t do that anymore.” [25] “I gave up smoking, which is a very difficult one to do and I done it just like that, so I'm sure if I can do that I can do anything.” [23] “Well, no, but I’ve lost weight. I’m sticking to the diet that the kidney doctor got me on. I’m drinking plenty of fluid. I’m not going to the bathroom as much as I was a month or two months ago, so that’s what gives me the thought that it’s going to get better. Because I’m gonna follow my [nephrologist’s] advice and better taken care of. I hope not. I hope they don’t [fail]…” [22] “You all ask like we took this by choice. We didn't have any control over this.” [25] “I could look on my machine and see sodium on there but I didn't know what I was looking at because nobody had never told me.” [25]
Adjusting to change	Particular stages of illness, such as diagnosis and CKD progression, required psychological adjustment	Psychosocial interventions could be used to promote adaption and acceptance	[20–26]	Low confidence	Minor concerns regarding methodology and relevance and moderate concerns regarding coherence and adequacy	“I don’t know what it’s like to be normal anymore, to feel normal.” [26] “I suppose in the back of your mind you think, ‘I don't want this’, cos you don't want any of it really. But then you just tell yourself, ‘well, you know you've got to deal with it, so you've got to make the best of what you've got.’” [23] “… I mean I go to music I go to art galleries and theatre. And er er so and films that’s one reason why I live in London. And so all of this is really important not talking unceasingly about my health.” [24] “When you’re first diagnosed it does feel like a death sentence... I’m like “35% [kidney function], so I’m dying right? So what do I expect? How long do I have?” [20] “I’m in denial now. It’s going again, listening to that man talking about, well, you know you’re a inch from being on the kidney dialysis machine. I don’t want to do that…” [22]
Containing fear of treatment and disease	Some patients were anxious and fearful regarding their future kidney treatment, with some also concerned about the impact of illness symptoms on their quality of life	Psychosocial support could create a space for patients to freely express anxiety and fears, as well as emphasise how quality of life can be maintained or improved	[20–23, 25, 26]	Moderate confidence	Minor concerns regarding methodology, coherence and relevance and moderate confidence regarding adequacy	“So I was concerned then because this… erm…. I had two pipes in here as well as this …erm…cut down the front…. I said will it be alright?” [21] “I have heard of too many people who have been on it [peritoneal dialysis] and almost died. I will not do it.” [25] “There’s no way I can go back to working where I used to, there’s no way I can stand on my feet for 8 h doing the heavy work I used to do, there’s all the retraining and going back into the workforce, plus trying to work out how I’m going to pay my bills, my rent.” [26] “The only thing is…for the pain, the stiffness, [residuals] from the stroke, I added a chiropractor. So, I think that’s what I wanted to say, exercise and all that different stuff like that. I do not basically exercise. And that’s why my walking and stuff like that, now that I want to get back into, you know, that helps a lot with illness and stuff.” [22] “And you know that the kidneys are going to be damaged again. That’s scary, I find.” [20]

#### Addressing the patient’s CKD-related educational needs

Patients expressed significant educational needs about their CKD across the studies [20–26]. Information appeared to empower patients, as they found education improved their quality of life [25], kept them informed [24], facilitated self-management [22] and reduced distress [20, 21]. However, some patients reported they lacked knowledge as it had not been shared [21] or that the information had been inadequate [20, 25]. Inconsistent or incorrect information was frustrating for patients [20, 21, 24, 25]. For example, whether it be about illness progression, treatment or diet, some patients noted finding such information confusing [21, 22, 24–26]. Patients’ sense of confusion could relate to the reported lack of time to discuss their questions and concerns about the information they received [21, 24, 25]. Information was sometimes given in the form of reading materials or videos [24, 25], which was recommended to be an addition to clinician-led education rather than the primary source of knowledge.

#### Supporting the patient’s relationships

Most studies explored the effects of different relationships in the patients’ lives [20, 21, 23–26], including with:

##### Healthcare professionals

Patients valued attentive, non-judgemental medical care and support in making decisions about renal replacement therapy [23, 26]. Some reported that they could trust that their healthcare team was looking after them, which helped patients to feel that they were being well monitored and looked after [20, 24]. However, some patients also shared that emotional and psychological support was lacking from their healthcare team [20, 26]. They could at times feel frustrated, disrespected, or de-valued, particularly if their symptoms were not necessarily CKD-related as they could feel ‘fobbed off’ with the recommendation to see another doctor [24]. Some reported distrusting their clinicians, or feeling that they were lied to [25]. This sense emerged from patients who had AKI stage 3D and felt their healthcare professionals had misled them in how their CKD would progress. The lack of supportive input from healthcare practitioners left patients feeling helpless [21]. These findings highlight the importance of healthcare professionals addressing their patients’ CKD-related educational needs as a way of improving their relationships. In doing so, patients may feel more valued, listened to, and respected and it may reduce risk of patients believing they were misled about how their kidney disease might progress.

##### Peers

Other patients with CKD were a significant source of support and information for patients [20, 21, 23, 25, 26]. Peers influenced decision-making for renal replacement therapy, as patients were made more aware of the treatment’s emotional and physical impact due to their interactions with patients who have more progressed CKD. Participants also found that peers helped them with coping and provided emotional support. Having someone who understood their experiences alleviated feelings of isolation and worry [23, 25, 26] and offered hope [20].

##### Family and friends

There appeared to be an undercurrent of the importance of family and friends’ support reported by patients [20, 22–26]. Family and friends sometimes played a role in supporting treatment management [22] and treatment decisions [23–25]. Family and friends were reported as actually making treatment decisions for the patient [25] or being supportive in creating a space for patients to share and receive thoughts about their options and talk about their illness [20, 23, 25]. Some patients were conscious of burdening their friends and family [23, 24]. Patients were aware of, and felt guilty about, their illness and symptoms' impact on their loved ones.

#### Honouring the patient’s need for control

Some patients reported they lacked control over their illness progression and choice in their treatment options [20, 21, 23, 25, 26]. The lack of control over their illness created a sense of helplessness [20, 21, 25, 26]. In contrast to these findings, other patients described confidence in their ability to adhere to future medical requirements and lifestyle changes, highlighting a potentially ameliorating role of patient self-efficacy [22, 23]. It was essential for patients to feel involved and respected when deciding on a treatment option [26]. Some patients did not understand the rationale behind the choices their healthcare team had made [24], while others felt they had no choice in their treatment at all [25].

#### Adjusting to change

Patients spoke of having to psychologically adapt to different stages of their illness [20–26]. Some patients described the experience of receiving a CKD diagnosis as traumatic and overwhelming, with some identifying that they experienced depression and anxiety in the early stages of their illness [26] or fearing they would die [20]. There was also a grieving process and, at times, denial of illness progression in which patients employed avoidant strategies to cope with uncertainty [20–22, 25]. Some patients appeared to adjust to their illness, although this may be due to being between critical points of their condition. This seemed to be facilitated by an internal process of accepting their situation, finding distractions that promoted their quality of life, and remaining positive and hopeful [20, 23, 24].

#### Recognising fear of treatment and disease

Some patients were anxious and fearful of their future treatment and disease progression [20–23, 25, 26]. While a few patients hoped renal replacement therapy would be minimally intrusive and that treatment would be flexible [23], others believed it would damage their quality of life and mental health [21, 23]. There was a sense of fear or apprehension of treatment outcome and the inevitability of CKD progression impacting their daily living [20, 21, 25]. In addition to fear of the future, several patients emphasised a present disease burden and how their symptoms negatively impacted them [22, 26]. Illness reportedly affected energy, financial security, social life, career and ability to have children [20, 26].

## Discussion

The present review synthesised qualitative findings on patients’ experiences of CKD without KRT to centre the patient voice and provide an additional perspective to previous quantitative findings. Thematic synthesis of seven studies identified five themes: addressing the patient’s CKD-related educational needs, supporting the patient’s relationships, honouring the patient’s need for control, adjusting to change, and recognising fear of treatment and disease. These findings can be used to develop therapeutic interventions for patients referred for psychosocial support.

Across the studies [20–26], patients highlighted their need for CKD-related education. Almutary and Tayyib [27] observed that knowledge was independently associated with self-management in a sample of patients who have CKD without KRT. These findings potentially reflect the empowering effects of CKD-related education. The quality and correct level of information appears to be critical—many participants in the studies reported finding the information they received confusing or that it was incorrect or insufficient [20–22, 24–26].

Patients’ relationships with their healthcare team, peers, and loved ones significantly impacted them. Some studies observed that patients described a lack of psychological support and could feel disrespected and not valued [20, 21, 24–26]. These findings echo another study with patients who have kidney failure, in which patients shared that they felt too embarrassed to talk about their emotional needs with their team [5]. The few who had the opportunity to attend a counsellor or psychologist reported that they found it challenging to access, and sessions felt time-limited.

Other patients or peers seemed to be a helpful resource for patients, offering insight from lived experience and emotional support. Qualitative interviews with patients who had different CKD stages and carers found that peer support was perceived as an opportunity to learn, reduce uncertainty and feel a sense of control [29]. Like the present review’s findings, participants were also conscious of burdening their loved ones and felt that they could rely on other patients who had CKD instead [29].

The themes of ‘honouring the patient’s need for control’, ‘adjusting to change’ and ‘recognising fear of treatment and disease’ reflect the importance of providing psychosocial support to help alleviate the disease burden in patients who have CKD without KRT. These themes reflect Hansen and colleagues’ [4] findings in their review of psychosocial needs in patients who have kidney failure. Patients who have kidney failure reported feeling a loss of freedom, autonomy and power, similar to some participants in the included studies, who shared a lack of control over their illness and a sense of helplessness [20, 21, 23, 25, 26]. Hansen and colleagues [4] also noted patients’ uncertainty about their mortality. In the present review, participants appeared to be concerned about their treatment and disease progression [20–23, 25, 26]. This shows that patients at very different stages of CKD experience fear and uncertainty in different ways, highlighting the need for psychosocial support to be offered to all patients.

The themes identified within this review are likely all related and could potentially impact on the psychological well-being of patients who have CKD without KRT. The themes of ‘addressing the patient’s CKD-related educational needs’ and ‘supporting the patient’s relationships’ are strongly interlinked. When sufficient time and space is given to respectfully educate the patient on their CKD, how they can manage it to maintain or improve their quality of life, and realistically manage expectations of disease progression in a compassionate way, the patient will likely feel more supported and well cared for by their healthcare team, improving trust in the relationship. Additionally, peers can be an invaluable source of information for patients and offering a means to create relationships with others who have CKD without KRT and who have progressed in their illness creates a helpful resource and psychological support for the patient. Educating the patient about their CKD, and potentially including family members and loved ones or offering family-specific education could also help to strengthen the patient’s personal relationships as increased awareness of CKD effects in the family might reduce the patient’s fears of burdening or that they cannot open up to their loved ones.

The themes of honouring the patient’s need for control, adjusting to change, and recognising fear of treatment and disease are strongly connected to one another. For example, if the patient has a strong fear of their future treatments and disease progression, they are likely to feel that they have little control over their disease and bodies. The patient will also likely have to adjust to CKD multiple times, from initial diagnosis, dietary changes, and adaptions to their daily lives to disease progression. Each change or adjustment could lead to a sense of loss of control or autonomy and increased fear or anxiety.

Addressing the patient’s CKD-related educational needs and supporting their relationships also relate to honouring the patient’s need for control, adjusting to change, and recognising fear of treatment and disease. Education will likely empower the patient and support them in their autonomy. An improved relationship with health care professionals or psychosocial interventions from the team will help the patient to grieve or adjust to their diagnosis or progression in their illness, and create a space for the patient to voice their fear.

### Implications, limitations, and future research

This review has many implications for practitioners. The findings highlight the importance of timing, and accurate, tailored CKD-related education delivered by clinicians for patients who have CKD without KRT. Patients should be offered the opportunity to raise questions and concerns and to feel there has been significant time spent with them. Groups could provide a valuable medium for providing CKD-related education and facilitating peer support. This could potentially improve patients’ relationships with healthcare staff as quality education may help the patient to feel valued and respected.

Additionally, clinicians can become aware of indications that the patient is distressed about burdening their family and offer education sessions that include a family member. Psychosocial practitioners can also offer further family work, and explore coping strategies within the wider system that will foster a sense of open communication and curiosity about the challenges that are faced (e.g. Behavioural Family Therapy, [29]). This will likely tie into individual interventions, where needed, that help patients adjust to their illness and manage expectations to improve their quality of life.

Psychosocial support could help the patient in the difficult stages of their disease, helping them adjust to advances in their condition or changes in their treatment. The so-called ‘Third Wave’ of Cognitive Behavioural Therapy, which to varying degrees aims to explore the meaning that patients ascribe to their experiences, and explores self-compassion, acceptance, and commitment to change, have all been evidenced to be useful in similar chronic ill-health groups (e.g. Acceptance and Commitment Therapy for diabetes patients [30]) and would be usefully explored further with this group, particularly as adjusting to change and the wish to be in control appear to be common difficulties for this group.

There are several limitations of the review. The review method deviated slightly from the pre-registered PROSPERO protocol. Initially, studies that mixed participants samples of patients who have CKD without KRT and patients who are in AKI stage 3D would be excluded for clarity. Except for Tong and colleagues’ article [26], this exclusion criterion was followed. This article was included as it identified CKD without KRT findings and data enough to be distinguished from other participants.

Five of the seven included studies are dated [21, 23–26], with two published in 2005. The review is limited in its ability to identify diverse and individualised needs of subgroups of patients due to the aim of synthesising and word count constraints, such as those that may be identified in young women [20], African Americans [22] and older patients [24]. Future qualitative research could investigate the specific psychological needs of marginalised patients.

All the included studies were conducted in Westernised countries with interviews and focus groups conducted in English, limiting the generalisability of the review findings to other cultures. Further qualitative research on the experiences of patients who have CKD without KRT in Asian and African countries and different languages would benefit any future systematic reviews as researchers could also explore similarities and differences cross-culturally.

## Conclusion

The present review aimed to synthesise qualitative research on the experiences of patients who have CKD without KRT, to provide additional insight into their potential psychosocial needs. Some patients reported finding information about CKD confusing or inadequate. Peers are identified as a helpful resource and could be capitalised on for an educational group intervention. Patients can feel conscious of burdening their friends and family. The review also observed several concerns for patients regarding the psychological effects of CKD without KRT. Patients appeared to feel helpless, as if they had lost control, had challenges adjusting and were fearful of the future. Therefore, the review highlights several psychosocial needs for patients who have CKD without KRT and potential areas for interventions.

## Supplementary Information

Below is the link to the electronic supplementary material.

Supplementary file1 (DOCX 17 kb)

## Data Availability

The datasets analysed during the current study are available from the corresponding author on reasonable request.
