# Supplementary material for: The psychosocial needs of patients who have chronic kidney disease without kidney replacement therapy: a thematic synthesis of seven qualitative studies
Source: J Nephrol. 2022 Sep 1;35(9):2251–67. doi: 10.1007/s40620-022-01437-3 (PMC9700594; doi:10.1007/s40620-022-01437-3)
Supplement: Supplementary file 1 — Supplementary file1 (DOCX 17 kb) [file 40620_2022_1437_MOESM1_ESM.docx]

Quality assessments of included articles, guided by COREQ

| **Authors** | **Strengths** | **Weaknesses** | **Reviewer Conclusions** |
| --- | --- | --- | --- |
| Andrew [19] | There was a brief description of the author (their occupation and training to date), as well as the methodological orientation (grounded theory) and some knowledge of the sample size. The author identified where the interviews take place and the table of themes they identified is clear. | There was no information on demographics, the interview schedule, how the pilot was used to influence the interview schedule. Additionally, the themes were just listed with no exploration or data. There is a presentation of a meta-theme but it is not based on clear evidence. As such, we cannot know if there is consistency between data and findings. | Article was excluded as findings were too thin to meaningfully interpret in context of the review. |
| Beanlands et al [20] | There was a detailed description of the recruitment process, setting of the focus group and analysis. The findings were justified with many quotations, demonstrating consistency between data and interpretations. The authors constructed a clear and interesting narrative. | The authors did not describe their theoretical underpinnings, relationship with participants or reflexive practices. They also did not share their justification for the sample size. | Article was included. |
| Iles-Smith [21] | There was a brief description of the author (qualification and experience). Themes appeared to be data-driven and quotes were used as evidence. | There is no demonstration of reflexivity or description of the authors’ relationship to the participants (this is important as the author is a renal nurse and we do not know if the participants were her patients). The author does not describe their recruitment strategy, extraction process, where the data was collected or if anyone assisted, the interview schedule and how themes ‘emerged’ or their theoretically underpinning. The author also did not identify which participant spoke a quote and as such, it is difficult to identify the diversity of opinions within the data (quotes could potentially rely on one participant). | We included this article as it provided rich findings despite methodological concerns. A CERQual assessment was used to consider the impact of these moderate methodological limitations on the findings. |
| Jennette et al. [25] | The authors provided detailed information on their methodology, describing their theoretical framework, when they ceased data collection, contexts of their focus groups and clearly stated analysis procedures. | The authors who collected data appeared to have a relationship with their patients as social workers but it is unclear what reflexivity they practised when engaging with the data. They did not identify drop-out rate or how many people did not agree to participate. The authors also did not identify which participant spoke a quote and as such, it is difficult to identify the diversity of opinions within the data (quotes could potentially rely on one participant). The authors also did not identify or describe any diverse cases or contradictions in data. | Article was included. |
| Lissanu et al [22] | The authors briefly reported on the characteristics of the interviewer. The characteristics might suggest the interviewers’ ability to build rapport with participants. There was a detailed description of the recruitment process and setting of the focus group. The findings were justified with many quotations, demonstrating consistency between data and interpretations. | The authors did not state their methodological orientation or theoretical underpinnings. They did not share their justification for the sample size. The authors describe developing a codebook for analysis and interrater reliability but do not clarify what method of analysis they used. The codebook was also originally informed by their interview schedule, which may have affected analysis. | Article was included. |
| Tong et al. [26] | The authors identified who recruited the participants, attendance rate of the focus groups and justified their aim for sample size. We felt they strongly reported their findings with many quotes as evidence. As the study included a mixed sample of pre-dialysis, dialysis and transplant patients, it was a significant strength that the authors identify what stage each participant was at. This facilitated inclusion of the article in the study. The authors include the characteristics of each focus group, including the emotional disposition, group dynamics and dominant focus of discussion. This provided interesting insight, although quotes are not matched with particular focus groups so they cannot be interpreted with the characteristics in mind. | The authors did not clarify who conducted the interviews and any relationships with patients were not explored. There is no evidence of reflexivity described. They did not state their conceptual methodological framework. The authors do not indicate how they assessed the emotional disposition or dynamics of the focus group. The authors also did not identify or describe any diverse cases or contradictions in data. | Article was included. |
| Tweed and Ceaser [23] | The authors clearly described their methodological orientation, participant selection and the setting of the interviews. The authors outlined number of data coders for analysis and a process for data triangulation. Findings were clearly reported and there appeared to be consistency between the data and findings. | The authors provided researcher details but any relationships or reflexivity processes were not described. They did not describe how many participants declined to participate and their sample size is not justified. The authors also did not identify or describe any diverse cases or contradictions in data. | Article was included. |
| Walker et al. [24] | There was a brief description of the lead author (credentials, occupation and training). There is also a description of how the researcher practised reflexivity. The methodological orientation and participant selection is described clearly. The authors outline the number of coders involved in analysis and provide a coding tree. Quotes are used as evidence for themes. There appears to be consistency between data and findings. | The researcher’s relationship to participants is not described, although they are described as a renal dietician. It is unclear of how they approached participants and if they were their patients. The authors also did not identify or describe any diverse cases or contradictions in data. | Article was included. |

CERQual Qualitative Evidence Profile

| **Summary of Review Finding** | **Studies contributing to the review finding** | **Methodological limitations** | **Coherence** | **Adequacy** | **Relevance** | ***CERQual assessment of confidence in the evidence*** | **Explanation of CERQual assessment** |
| --- | --- | --- | --- | --- | --- | --- | --- |
| **1.** Patients felt that they did not receive sufficient information about their healthcare, or that it was overly confusing. They had additional questions left unanswered and wanted additional time with their healthcare team to discuss their concerns. Communication training and group psychoeducation could support educational needs. | [20-26] | Minor methodological limitations (six studies with minor and one study with moderate methodological concerns) | Minor concerns about coherence | Minor concerns about adequacy (seven studies that together offered moderately rich data) | Minor concerns about relevance | Moderate confidence | Minor concerns regarding methodological limitations, coherence, adequacy and relevance. |
| **2. *Healthcare professionals.*** Patients valued attentive, non-judgemental medical care but sometimes found this was lacking, creating a sense of distrust and disrespect.  ***Peers.*** Peers were a supportive educational and emotional resource to patients, highlighting the potential effectiveness of group support for patients at a pre-dialysis stage.  ***Family and friends.*** Support from family and friends was important to patients, influencing their illness-related decision-making. Patients felt conscious of burdening their loved ones with their illness. | [20, 22-26] | Minor methodological limitations (six studies with minor and one study with moderate methodological concerns) | Minor concerns about coherence | Moderate concerns about adequacy (seven studies that together offered moderately rich data; one study that expressed significant frustration with healthcare providers; studies did not significantly describe data relating to personal relationships) | Minor concerns about relevance | Moderate confidence | Minor concerns regarding methodological limitations, coherence and relevance and moderate concerns regarding adequacy. |
| **3.** At times, patients felt like they lacked control of their illness and treatment, creating a feeling of helplessness. Feeling listened to, educated on their treatment and addressing symptoms using psychosocial support could foster self-efficacy. | [20, 21, 23, 25, 26] | Minor methodological limitations (four studies with minor and one study with moderate methodological concerns) | Minor concerns about coherence(some concerns about the fit between data and review findings) | Minor concerns about adequacy (five studies that together offered moderately rich data) | Minor concerns about relevance | Moderate confidence | Minor concerns regarding methodological limitations, coherence, adequacy and relevance. |
| **4.** Particular stages of illness, such as diagnosis and progression towards dialysis, required psychological adjustment, offering an opportunity to promote adaption and acceptance. | [20-26] | Minor methodological limitations (six studies with minor and one study with moderate methodological concerns) | Moderate concerns about coherence (Some concerns about the fit between data and review findings) | Moderate concerns about adequacy (studies offered thin data with some overlap between containing fear of treatment and disease theme) | Minor concerns about relevance | Low confidence | Minor concerns regarding methodology and relevance and moderate concerns regarding coherence and adequacy. |
| 5. Some patients were anxious and fearful regarding their future renal treatment, with some also concerned about the impact of illness symptoms on their quality of life. | [20-23, 25, 26] | Minor methodological limitations (five studies with minor and one study with moderate methodological concerns) | Minor concerns about coherence (some concerns about the fit between data and review findings) | Minor concerns about adequacy (six studies that together offered moderately rich data) | Minor concerns about relevance | Moderate confidence | Minor concerns regarding methodology, coherence and relevance and moderate confidence regarding adequacy |
